# Attentional spreading to task-irrelevant object features: experimental support and a 3-step model of attention for object-based selection and feature-based processing modulation

**DOI:** 10.3389/fnhum.2014.00414

**Published:** 2014-06-10

**Authors:** Detlef Wegener, Fingal Orlando Galashan, Maike Kathrin Aurich, Andreas Kurt Kreiter

**Affiliations:** Center for Cognitive Science, Brain Research Institute, University of BremenBremen, Germany

**Keywords:** reaction times, object-based attention, feature-based attention, attention model, task difficulty

## Abstract

Directing attention to a specific feature of an object has been linked to different forms of attentional modulation. Object-based attention theory founds on the finding that even task-irrelevant features at the selected object are subject to attentional modulation, while feature-based attention theory proposes a global processing benefit for the selected feature even at other objects. Most studies investigated either the one or the other form of attention, leaving open the possibility that both object- and feature-specific attentional effects do occur at the same time and may just represent two sides of a single attention system. We here investigate this issue by testing attentional spreading within and across objects, using reaction time (RT) measurements to changes of attended and unattended features on both attended and unattended objects. We asked subjects to report color and speed changes occurring on one of two overlapping random dot patterns (RDPs), presented at the center of gaze. The key property of the stimulation was that only one of the features (e.g., motion direction) was unique for each object, whereas the other feature (e.g., color) was shared by both. The results of two experiments show that co-selection of unattended features even occurs when those features have no means for selecting the object. At the same time, they demonstrate that this processing benefit is not restricted to the selected object but spreads to the task-irrelevant one. We conceptualize these findings by a 3-step model of attention that assumes a task-dependent top-down gain, object-specific feature selection based on task- and binding characteristics, and a global feature-specific processing enhancement. The model allows for the unification of a vast amount of experimental results into a single model, and makes various experimentally testable predictions for the interaction of object- and feature-specific processes.

## Introduction

The term attention is widely used to paraphrase specific modulations in the representation of task-relevant sensory information. While it suggests the assumption of a homogenous process, attention research has revealed many different aspects of attentional modulation, both in terms of neuronal mechanisms and behavior, and not all of these results turned out to be easily compatible.

Most confidence has been obtained for processing the attended information. Studies investigating the influence of attention on neuronal responses revealed a multitude of effects. For instance, directing attention to the motion of a stimulus, in terms of direction and speed, locally increases the firing rate (Treue and Maunsell, 1996) and the gamma power of the local field potential (Khayat et al., 2010) of neurons in motion-sensitive mediotemporal (MT) area, causes shrinkage of receptive fields around the attended stimulus (Womelsdorf et al., 2006a), and increases stimulus selectivity of single neurons (Wegener et al., 2004). As a consequence of attentional modulation, task-relevant motion changes are represented with shorter latency, and reaction times (RTs) become faster (Galashan et al., 2013). Corresponding findings have been obtained in other visual areas for features like color and form (McAdams and Maunsell, 1999; Reynolds et al., 1999; Fries et al., 2001; Taylor et al., 2005; Sundberg et al., 2012).

Less clear than the enhanced processing of the selected information is the processing fate of currently task-irrelevant, unattended information. For instance, if attention is directed to the motion of a colored object, what about processing of the target object’s color, or motion information at other objects? In the framework of object-based attention theory, objects are considered the natural “units of attention”, and attending a certain object feature has been shown to cause spreading of attention to other features of that object, thus promoting selection of the entire object (Duncan, 1984; O’Craven et al., 1999; Blaser et al., 2000; Scholl, 2001; Rodríguez et al., 2002; Schoenfeld et al., 2003; Wannig et al., 2007; Ernst et al., 2013). If taken literally, object-based attention requires restriction of any response modulation to features at the attended object by definition, without spreading to features at other objects. However, psychophysical, imaging, and electrophysiological studies showed that attending towards a certain object feature is associated with enhanced processing of that feature throughout the visual field (Rossi and Paradiso, 1995; Treue and Martínez Trujillo, 1999; McAdams and Maunsell, 2000; Saenz et al., 2002; Arman et al., 2006; Müller et al., 2006; Serences and Boynton, 2007). In addition, various recent studies indicated that selection of a single target-object feature may result in suppression of other, task-irrelevant features of that object (Fanini et al., 2006; Nobre et al., 2006; Cant et al., 2008; Polk et al., 2008; Wegener et al., 2008; Serences et al., 2009; Taya et al., 2009; Xu, 2010; Freeman et al., 2014).

**Figure 1:**
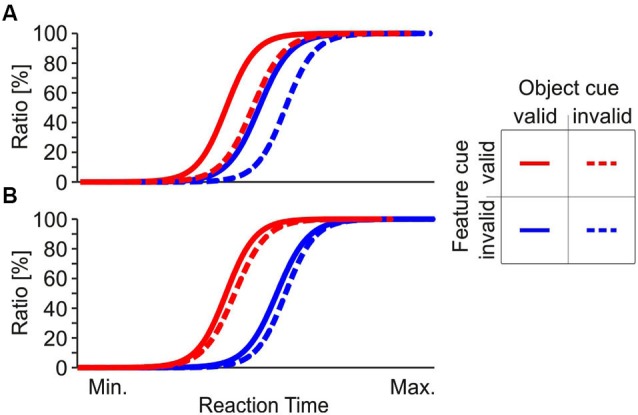
**Hypothetical RT distributions. (A)** Cumulative RT distribution compatible with an object-based attention approach. Task-irrelevant features are associated with faster RTs if they belong to the attended object, as indicated by a leftward shift of the RT distributions for invalidly cued features. **(B)** Cumulative RT distribution incompatible with a strict object-based attention approach. Here, object cueing is ineffective (i.e., a valid object cue has no influence or has an equal influence at both objects) and RTs are influenced solely by the feature cue, as indicated by overlaying RT distributions for corresponding feature cue conditions.

These results might be perceived as conceptually contradictory in some cases, and apparently conflicting in others. Understanding the underlying attentional mechanisms will critically depend on investigating the interaction of different forms of attention. This issue has been addressed by a surprisingly small number of studies (Boehler et al., 2011; Kravitz and Behrmann, 2011; Lustig and Beck, 2012). Since many of the above cited studies used the basically same behavioral requirement of object-feature directed attention, we performed two psychophysical experiments to further investigate the interaction and potential co-existence of feature- and object-based attention. To this end, we used stimulus and task conditions similar to those previously utilized for demonstrating object- and feature-based attention (Schoenfeld et al., 2003, 2014; Müller et al., 2006). We used RT as a measure for attention-dependent processing, and studied attentional spreading along both the object and feature domain in parallel. We presented two superimposed random dot arrays at fixation, having either motion in opposite direction but the same color, or having different colors but the same motion direction. Subjects were asked to make speeded responses to changes of either speed or color at one of the two objects, and attention was directed using cues indicating the object and the feature for which the change was to occur, with 75% validity. Figure 1 shows two hypothetical patterns of cumulative RT distributions. In Figure 1A, fastest responses occur if both the feature and the object-cue dimension are correct, and slowest responses occur if both are incorrect. RTs are in-between if only one of the two cue dimensions is correct. Since responses to the unattended feature are faster if they occur at the attended object (straight blue line shifted to the left as compared to the dashed blue line) this result pattern suggests an object-based benefit for the unattended feature. In Figure 1B, only the feature dimension of the cue is effective, but the object dimension has no impact. Such an RT pattern is in favor of a pure feature-based attentional modulation, since RTs solely depend on attention directed to the feature, with no differences between attended and unattended object.

Our findings suggest that both feature- and object-specific attentional effects are evident at the same time. The results confirm that attending a single target-object feature is accompanied by co-selection of other, task-irrelevant features of the same object. However, they also show that this modulation is not restricted to the selected object but instead, spreads towards the unattended object. We suggest a simple, physiologically plausible 3-step model of attention to unify findings from object-based and feature-based attention theory in a single framework. Preliminary results have previously been published in abstract form (Wegener et al., 2009, 2010).

## Material and methods

### Subjects

The study was conducted with eight naïve female participants (mean age: 25.8 years). All subjects had normal or corrected-to-normal vision, as approved by the Freiburg Visual Acuity Test (Bach, 1996), and gave their written informed consent. The study conformed to the Code of Ethics of the World Medical Association (Declaration of Helsinki) and was approved by the University’s ethics committee.

### Visual stimulation and task

**Figure 2:**
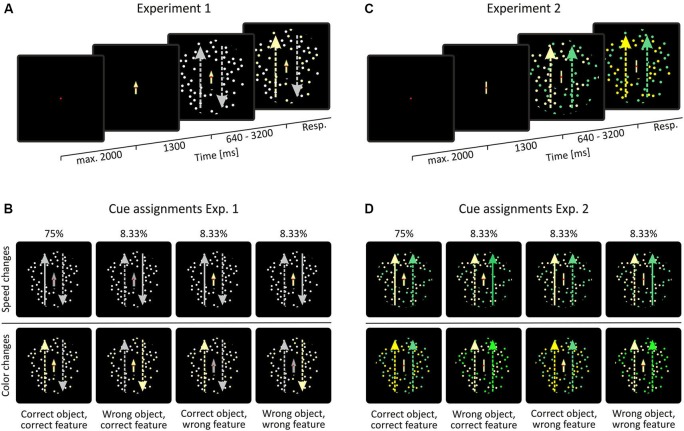
**Behavioral task and visual stimulation. (A, C)** In both experiments, stimuli consisted of two superimposed, doughnut-shaped random-dot patterns (RDPs), placed at the center of the screen. Motion coherence was 100% in both of the patterns. In Experiment 1, motion was the object-defining feature and color was the shared feature (i.e., motion was in opposite direction and color was the same), whereas the opposite was true for Experiment 2 (i.e., motion direction was the same but color differed). **(B, D)** Cue assignments in experiment 1 and 2. Long, lateral arrows represent the features of the RDPs: arrow direction indicate RDP motion direction, dotted lines indicate normal speed, straight lines indicate increased speed, and arrow color indicates RDP color. These arrows are shown for illustration purposes only and were not part of the display. Note that stimulus colors were chosen for illustration purposes only. Actual colors used in the experiment were slightly different and color changes were less obvious as compared to the figure.

The behavioral task consisted of a feature-change detection paradigm, as outlined in Figure 2. Stimuli consisted of two superimposed, doughnut-shaped random dot patterns (RDPs) presented at the center of the screen with the fixation point and the cue being located in the inner notch of the stimulus. Stimuli had a diameter of 6.34° with the notch being 1.9° in diameter. Each RDP consisted of 50 dots with a maximal lifetime of 200 ms. Dot positions within the RDP were calculated as to never overlap each other, thus resulting in an individual dot’s lifetime of mostly less than 200 ms. In Experiment 1 (Figure 2A), RDPs possessed coherent motion in opposite directions along the vertical meridian, at a constant speed of 2.54°/s. Color was the same for both RDPs. In case of a speed change, speed increased by 50%, in case of a color change, color switched from white to pale yellow. Speed and color change trials were cued by an arrow that was either gray (in case of a presumed speed change) or pale yellow (in case of a presumed color change). The orientation of the arrow indicated the direction of motion of the RDP on which the change was to occur. Cues had a validity of 75%. In case of an incorrect cue, the cue either (i) indicated the correct object, but the wrong feature to be changed; or (ii) the correct feature, but the wrong object; or (iii) was wrong in both respects (Figure 2B). In Experiment 2, RDPs consisted of isoluminant yellow and green dots but had coherent motion in the same direction (Figure 2C). In case of a speed change, the target object’s speed again increased by 50%, in case of a color change dot color was getting slightly more intense. Color changes were matched to be as equally difficult to detect as those in Experiment 1, as confirmed in independent test trials with other subjects. Cues of Experiment 2 consisted of arrows (in case of a presumed speed change) or bars (in case of a presumed color change). Cue color (yellow or green) indicated the object on which the change was to occur (Figure 2D).

Subjects sat 45 cm in front of a 22 inch monitor (NEC Multisync FB2111 SB, NEC Display Solutions, Munich, Germany), with their head stabilized by a head-chin rest. Stimuli were generated on a Pentium computer with an NVidia Quadro NVS graphics card and were displayed on a dark background with a resolution of 1280 × 1024 pixels, at 100 Hz horizontal refresh rate. Eye position was monitored using a CCIR Monochrome Camera (DMK 83 Micro/C, The Imaging Source, Bremen, Germany) and a custom-made remote videooculography system.

Each trial started with the appearance of a red fixation point in the center of the screen. Subjects initiated the trial by pressing a handheld button and keeping it pressed until a response was required. Following trial initiation, the cue appeared in the center of the screen with the fixation point superimposed on it, and remained visible throughout the trial. After a delay period of 1300 ms the two RDPs were displayed. Following RDP onset, one of the patterns changed either speed or color at one of nine possible points in time, separated by 320 ms between 640 ms and 3200 ms. Subjects were required to respond to any change as quick as possible, but in any case within a response interval of maximally 1000 ms, by releasing the button. Note that they were only required to detect but not to discriminate the change. Subjects were given immediate auditory feedback about their RTs by using sinus tones of different pitch. Very fast RTs were indicated by a different, especially pleasant tone. Divergence of the eye position by more than 1° from the fixation point, release of the button prior to any change (false alarm), or absence of a response 1 s after the change (miss) caused immediate termination of the trial.

Per experiment, data were obtained within nine consecutive blocks, with no more than two blocks per day. Each block consisted of 96 trials, i.e., 48 trials per feature change condition. Speed and color change as well as correctly and incorrectly cued trials were fully interleaved with the order randomly chosen by the stimulation program. Prior to both experiments, subjects were given one block to familiarize them with the task and stimuli.

### Data analysis

Data were analyzed with custom-written scripts and the Statistics Toolbox in Matlab 7.13 (The MathWorks, Natick, MA). Trials in which the button was released at or before 200 ms after a feature change were counted as false alarms. Performance was calculated as the percentage of correct responses from the sum of correct responses, false alarms, and misses. RT analysis was performed separately for speed and color change trials. To avoid influences of day-by-day variations in RT and to allow for comparing RTs across experiments, each speed and color change RT was normalized by dividing through the mean RT of all speed and color change trials, respectively, of the corresponding block. Group RTs were calculated as the average of the median normalized RTs per subject and cue condition. Feature- and object-cue effects were analyzed by 2-way ANOVA using the factors feature cue (valid, invalid) and object cue (valid, invalid). *Post-hoc* tests were conducted with two-tailed paired *t*-tests. All tests were performed on a 95% significance level.

## Results

### Behavioral data

Subjects performed a total of 864 trials in each of the experiments, registered within nine consecutive blocks distributed over usually 5 days. Eye movements exceeding 1° from central fixation or eye blinks resulted in termination of 2.5% of all trials. Excluding these fixation errors, mean performance was 92.8 ± 1.5% in Experiment 1 and 92.9 ± 1.3% in Experiment 2, and was very similar between speed and color change trials (range: 92.1–93.6%). Regarding practicing effects over blocks, mean performance in Experiment 1 increased slightly during the course of the experiment but did not show significant variations, whereas in Experiment 2, performance in the first session was worse than in some subsequent sessions, as revealed by block-wise comparison of the percentage of successful trials by means of 1-way ANOVA and Bonferroni’s Multiple Comparison Test (Experiment 1: *F*_(8,63)_ = 1.8, *p* = 0.094; Experiment 2: *F*_(8,63)_ = 4.2, *p* = 0.0005). Considering the relevant behavioral measure of this study, we found very similar RTs across blocks for both correctly cued speed and color changes in both experiments, with no significant difference between blocks (Experiment 1: speed: *F*_(8,63)_ = 0.24, *p* = 0.981, color: *F*_(8,63)_ = 0.85, *p* = 0.563; Experiment 2: speed: *F*_(8,63)_ = 1.1, *p* = 0.378, color: *F*_(8,63)_ = 0.8, *p* =0.609). For optimal comparability between speed-change and color-change trials and between experiments, we normalized all speed-change RTs of a subject to the mean speed-change RT of the respective experimental block, and proceeded accordingly for color-change trials. All results reported in this paper also hold true for absolute RTs.

### Experiment 1—Objects defined by motion direction

**Figure 3:**
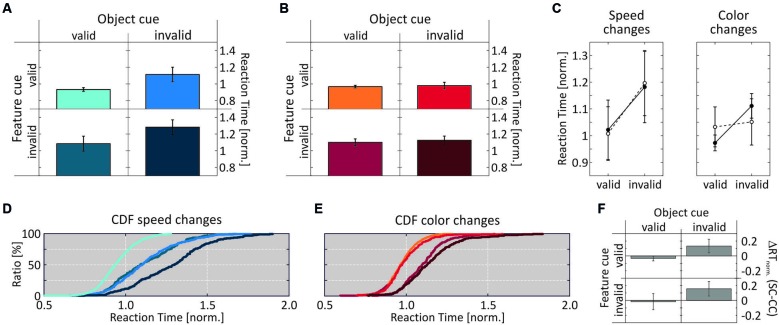
**Results of Experiment 1. (A)** RT results for speed-change and **(B)** color-change detection as a function of validity of the two cue dimensions. Per cueing condition, bars represent the mean over the median normalized RTs of all subjects. **(C)** Mean normalized RTs for the factors feature cue (black circles) and object cue (open circles) as a function of cue validity. For the feature cue, plotted values represent the row mean of the data shown in A, B and for the object cue they represent the column mean. **(D, E)** Cumulative distributions of normalized RTs. Line colors correspond to cue conditions as in **(A, B)**. **(F)** Speed- and color-change difference of mean normalized RTs for corresponding cue conditions. Error bars indicate SD throughout the figure.

Figure 3 shows the RT results for Experiment 1, when objects were defined by motion direction and color was the shared feature. For speed changes, mean normalized RTs were fastest when both the feature and the object cue were correct (0.932 ± 0.022), and slowest when both were incorrect (1.281 ± 0.088). When either the feature or the object cue dimension was correct and the other cue dimension was incorrect, RTs were in-between (1.112 ± 0.087 and 1.083 ± 0.09, respectively; Figure 3A). For comparison with the literature, Table 1 lists absolute RTs. A 2-way ANOVA with the factors feature cue (valid, invalid) and object cue (valid, invalid) revealed highly significant effects of both factors (feature cue: *F*_(1,7)_ = 34.3, *p* < 0.0001; object cue: (*F*_(1,7)_ = 47.8, *p* < 0.0001), and no interaction (*F*_(1,7)_ = 0.121, *p* = 0.73; Figure 3C, left). *Post-hoc* two-tailed *t-tests* confirmed these results by showing highly significant effects of the feature cue at both the correctly (*p* = 0.0023) and incorrectly cued object (*p* = 0.0036), as well as highly significant effects of the object cue for both correctly (*p* = 0.0015) and incorrectly cued features (*p* = 0.0025). Both cue dimensions were about equally effective as revealed by no differences between the two conditions if only one of the two cue dimensions was correct and the other incorrect (*p* = 0.6367). The corresponding cumulative distributions of RTs are shown in Figure 3D, revealing a close similarity with the hypothetical pattern of distributions to illustrate an object-cue benefit, as shown in Figure 1A. The critical comparison here is the distribution of RTs for the two conditions using invalid feature cues, showing a clear leftward shift of the RT distribution if the unattended speed change occurred at the attended object as compared to the unattended object.

**Table 1:** **Absolute mean RTs ± SD [ms] for the four different cueing conditions of Exp. 1 and Exp. 2**.

		Object cue Exp. 1		Object cue Exp. 2	
		valid	invalid	valid	invalid
Feature cue: Speed Change	valid	385 ± 43	458 ± 50	366 ± 49	377 ± 56
	invalid	447 ± 65	533 ± 52	438 ± 64	454 ± 67
Feature cue: Speed Change	valid	343 ± 39	352 ± 60	345 ± 35	389 ± 45
	invalid	384 ± 53	404 ± 58	406 ± 54	455 ± 78

We next investigated whether this pattern of results also holds true for the detection of color changes. As for speed changes, we found fastest RTs for fully correctly cued trials (0.967 ± 0.016), and slowest RTs for fully incorrectly cued trials (1.124 ± 0.05). Yet, for the two conditions having one incorrect cue dimension, RTs were almost exclusively determined by the validity of the feature cue: if the feature cue was correct, RTs at the uncued object (0.979 ± 0.039) were close to those at the cued object, and if the feature cue was incorrect, RTs at the cued object were close to those at the uncued object (1.099 ± 0.041; Figure 3B). A 2-way ANOVA revealed a highly significant effect of the factor feature cue (*F*_(1,7)_ = 102.9, *p* < 0.0001), but no effect of the factor object cue (*F*_(1,7)_ = 1.8, *p* = 0.185), and no interaction (*F*_(1,7)_ = 0.195, *p* = 0.662; Figure 3C, right). *Post-hoc* two-tailed *t*-tests showed a significant difference between the two conditions having only one correctly cued dimension (*p* = 0.0002), but no differences between the conditions having a correct or an incorrect feature cue at either the cued (*p* = 0.473) or the uncued (*p* = 0.111) object. Thus, for color changes in Experiment 1 the results were different from those of speed changes, as reflected by a pattern of cumulative RT distributions (Figure 3E) similar to those shown in Figure 1B, illustrating a strict feature-based modulation of RTs. Moreover, comparing RTs in response to color changes with those in response to speed changes revealed very similar RTs if the object cue was correct, but also significantly shorter color-change RTs if it was incorrect (correctly cued features: *p* = 0.0045; incorrectly cued features: *p* = 0.0026; Figure 3F). Hence, while results for speed changes confirmed predictions of object-based attention theory regarding a same-object benefit, those for color changes were more in line with feature-based modulation.

### Experiment 2—Objects defined by color

The failure to find a same-object benefit for color-change detection in Experiment 1 could be due to either absent attentional co-selection of the task-irrelevant feature at the cued object, or alternatively, to a spreading of feature-dependent attention towards the uncued object. Both possibilities potentially result in RTs being not different at the cued or uncued object. As a third alternative, the dichotomy in speed- and color-change detection may represent a general difference in attention-dependent processing of the two features. We tested between these alternatives by performing another experiment using objects differing in color but not motion direction. We hypothesized that a general difference in speed- and color-change detection should preserve the pattern of RT distributions found in Experiment 1, whereas these should be inverted (i.e., a same-object benefit now for color but not motion) if one of the former alternatives was true.

**Figure 4:**
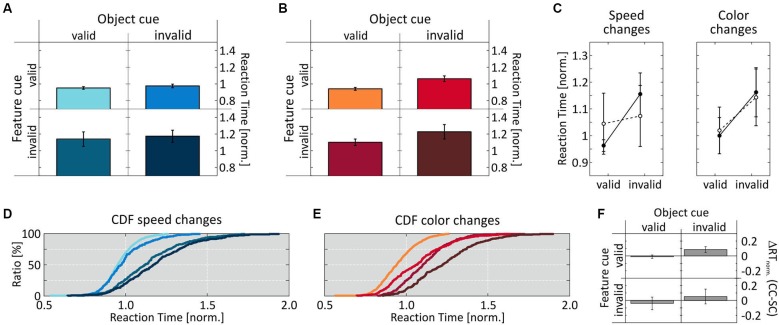
**Results of Experiment 2. (A, D)** RT results for speed-change and **(B, E)** color-change detection as a function of validity of the two cue dimensions. **(C)** Mean normalized RTs for speed- and color-change detection as a function of object-cue validity. **(F)** Speed- and color-change difference of mean normalized RTs for corresponding cue conditions. Conventions as in Figure 3.

Figure 4 illustrates that the results of Experiment 2 were exactly opposite to those of Experiment 1. For speed changes, we now obtained a pattern of RT distributions similar to those for color changes in Experiment 1, with no same-object benefit: RTs were similarly fast at both the correctly and incorrectly cued object (0.951 ± 0.016 and 0.975 ± 0.022, respectively) when the feature cue was correct, and similarly slow when the feature cue was incorrect (1.14 ± 0.087 and 0.172 ± 0.072, respectively) (Figures 4A,D). A 2-way ANOVA revealed a significant influence of the factor feature cue (*F*_(1,7)_ = 88.8, *p* < 0.0001), but not of the factor object cue (*F*_(1,7)_ = 2.0, *p* = 0.168), and no interaction (*F*_(1,7)_ = 0.051, *p* = 0.823; Figure 4C, left). *Post-hoc* analysis confirmed the feature cue effect at both the cued and the uncued object (*p* = 0.001 and *p* < 0.001, respectively).

In contrast, for color-change detection we now found a clear same-object benefit, thus resembling the results for speed changes in Experiment 1: RTs were again fastest when both cue dimension were correct (0.94 ± 0.017), and slowest when both were incorrect (1.23 ± 0.087). If only one feature dimension was correct and the other incorrect, RTs were in-between (correct feature cue: 1.061 ± 0.034; correct object cue: 1.1 ± 0.04), indicating an influence of both cue dimensions (Figures 4B,E, cf. Table 1 for absolute RT values). Accordingly, performing a 2-way ANOVA revealed a significant influence of both factors (feature cue: *F*_(1,7)_ = 80.6, *p* < 0.0001 ; object cue: *F*_(1,7)_ = 46.8, *p* < 0.0001), and no interaction (*F*_(1,7)_ = 0.017, *p* = 0.897; Figure 4C, right). *Post-hoc* *t-tests* confirmed this by showing significantly shorter RTs between correctly and incorrectly cued features at both the correctly and incorrectly cued object (*p* < 0.0001 and *p* = 0.0031, respectively), and significantly shorter RTs depending on the validity of the object cue for both correctly and incorrectly cued features (both *p* < 0.0001). Different to Experiment 1, however, comparing the two conditions having only one correctly cued dimension revealed a slightly, but significantly higher influence of the feature cue (*p* = 0.021). Comparing the cue effects for speed and color changes again revealed very similar RTs at the cued object, but slightly faster RTs for speed changes at the uncued object, which were significant for correctly cued features (*p* = 0.0004) (Figure 4F).

### Comparison of speed- and color-change detection across experiments

**Figure 5:**
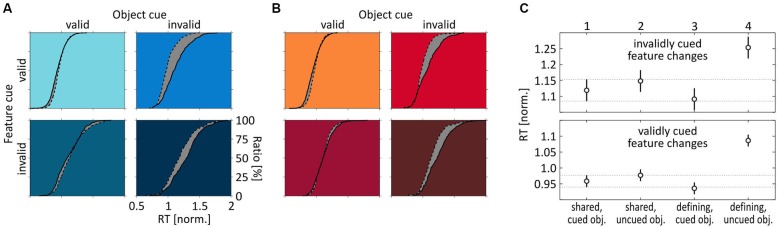
**Comparison of feature-change detection across Experiments 1 and 2. (A)** RT distributions for speed-change and **(B)** color-change detection under the four different cue conditions, depending on whether the feature was object-defining (straight line) or shared (dashed line). Scaling of axes is identical for all subplots, as indicated in the right bottom panel of **(A)**. **(C)** Mean normalized RTs for shared and object-defining features, separately for invalidly (top) and validly (bottom) cued features. RTs are pooled across both speed and color-change trials. Error bars indicate 95% confidence intervals for each mean, and the horizontal lines plot the 95% CI estimated for changes of the shared feature at the cued object, as a reference. Numbers on top of upper x-axis indicate stimulus and cue condition for reference to statistical comparisons summarized in Table 2.

Experiment 2 showed that the existence or absence of a same-object benefit is not due to a general difference between speed- and color-change detection. Thus, we next investigated whether it is caused by either absent attentional co-selection of the task-irrelevant feature at the attended object, or alternatively by attentional spreading of feature-dependent attention towards the unattended object. To this end, we analyzed speed- and color-change detection across experiments, i.e., we compared RTs in response to a feature change when it was the unique, object-defining feature vs. when it was the shared one. We found that speed and color changes provided an essentially identical pattern of results (Figures 5A,B). At the cued object, RT distributions were basically indistinguishable between Experiments 1 and 2, i.e., they were about the same independent of whether the feature was object-defining or shared. In contrast, at the uncued object we observed a prominent leftward shift of the RT distribution when subjects responded to a change of the shared feature, regardless of whether this was speed or color, or whether the feature was correctly or incorrectly cued. These findings allow for two important conclusions regarding attentional spreading: First, since RTs at the cued object where equal for shared and object-defining features, the task-irrelevant shared feature received the same attentional modulation as the object-defining feature (for which a same-object benefit was evident for both speed and color changes), thus indicating attentional co-selection of the task-irrelevant target-object feature independent of its relevance for defining or selecting the object. Second, since RT distributions for shared features were consistently shifted to the left at the uncued object, attentional modulation of shared features was not restricted to the target but spread towards the task-irrelevant object, resulting in a failure to find a same-object benefit for shared features in the previous analyses. Hence, attending towards a single feature of a target object resulted in co-selection of another, task-irrelevant feature of that object. Yet, the underlying attentional process was not restricted to the selected object, but included enhanced processing of that irrelevant feature at another, irrelevant object.

**Table 2:** **Statistical results for comparing change detection at the cued and uncued object, separately for object-defining and shared features, and depending on the feature cue being either validly (upper half) or invalidly (lower half) cued**.

	Stimulus conditions	Confidence interval		Mean difference	Significantly different
		Lower bound	Upper bound		
Feature cue valid	1 vs. 2	−0.0570	0.0201	−0.0185	no
	1 vs. 3	−0.0159	0.0612	0.0226	no
	1 vs. 4	−0.1663	−0.0892	−0.1277	yes
	2 vs. 3	0.0026	−0.0796	0.0411	no
	2 vs. 4	−0.1478	−0.0708	−0.1093	yes
	3 vs. 4	−0.1889	−0.1119	−0.1504	yes
Feature cue invalid	1 vs. 2	−0.0998	0.0415	−0.0291	yes
	1 vs. 3	−0.0428	0.0985	0.0279	no
	1 vs. 4	−0.2050	−0.0637	−0.1344	yes
	2 vs. 3	−0.0137	0.1276	0.0570	no
	2 vs. 4	−0.1759	−0.0346	−0.1053	yes
	3 vs. 4	−0.2329	−0.0916	−0.1622	yes

Stimulus condition numbers correspond to those introduced in Figure 5C.

This conclusion is supported by a balanced one-way ANOVA using data for shared and object-defining features at both the cued and the uncued object, pooled over speed- and color-change trials from both experiments (Figure 5C). For both validly and invalidly cued features, ANOVAS indicated significant differences between the four cue conditions (uncued features: *F*_(3, 60)_ = 15, *p* < 0.0001; cued features: *F*_(3, 60)_ = 44.67, *p* < 0.0001). For testing individual conditions, we applied a Bonferroni correction for multiple comparisons and regarded conditions as being significantly different if the confidence interval did not include 0 for alpha errors of 0.05. Mean differences and corresponding lower and upper bounds of confidence intervals are summarized in Table 2. For uncued changes of the object-defining feature, we found significantly faster RTs at the cued object, confirming the same-object benefit as described previously by analyzing speed- and color change trials individually. However, changes of the shared feature were statistically not different from those of the object-defining feature at the cued object, independent of the object on which they occurred. Even more, they were consistently faster than RTs to changes of the object-defining feature at the uncued object. Thus, a feature that was fully irrelevant to select the object received the same attentional modulation than another one that was obligatorily required for object selection, and this attentional modulation spread towards the task-irrelevant object. A similar pattern of results was found for correctly cued feature changes. Again, changes of the object-defining feature were not only significantly slower at the uncued object as compared to the cued one, but also as compared to changes of the shared feature, regardless of whether these occurred at the cued or the uncued object. The only difference to the former analysis for invalidly cued feature changes was that changes of the shared feature at the uncued object were slightly but significantly slower than those of the object-defining feature at the cued object. Thus, statistically testing confirmed our previous conclusion that the absence of a same-object benefit for shared features was not due to absent attentional modulation of that feature but caused by spreading of attention from the co-selected irrelevant object feature to the same feature at the unattended object.

## Discussion

Object feature-directed attention (OFDA) has been associated with co-selection as well as suppression of task-irrelevant target-object features, and with a global spreading of attention towards distant objects sharing the attended feature (for review: Olson, 2001; Scholl, 2001; Maunsell and Treue, 2006; Carrasco, 2011; Chen, 2012; Lee and Choo, 2013). Several factors influencing whether features are processed independently or integrated over objects were postulated, including stimulus characteristics (Vecera and Farah, 1994), the spatial extent of attention (Lavie and Driver, 1996), the need of attentional shifts (Lamy and Egeth, 2002), and task demands (Mayer and Vuong, 2012). Co-selection of task-irrelevant object features has been taken as evidence for object-based attention (Duncan, 1984; O’Craven et al., 1999; Blaser et al., 2000; Rodríguez et al., 2002; Schoenfeld et al., 2003; Wannig et al., 2007), while suppression of task-irrelevant features and global enhancement of the attended feature has been attributed to feature-based attention (Rossi and Paradiso, 1995; Treue and Martínez Trujillo, 1999; Saenz et al., 2002, 2003; Martínez Trujillo and Treue, 2004; Fanini et al., 2006; Nobre et al., 2006; Polk et al., 2008; Wegener et al., 2008; Gál et al., 2009; Serences et al., 2009). Yet, it is an open question whether the different effects observed with OFDA represent different attention mechanisms of which one dominates the other depending on task and stimulus constraints, or whether they represent distinct, potentially co-existing states of a single attention mechanism. The current study provides evidence for the latter possibility by demonstrating that object- and feature-specific effects of attention are not mutually exclusive but co-exist, as expressed by effective attentional modulation of task-irrelevant, co-selected target-object features at non-target objects. To conceptualize our findings, we propose a 3-step model of attention consisting of object-specific selection of features due to binding and grouping dynamics and a subsequent global, object- and space-independent modulation of those selected features. Upstream to this, a task-dependent, weighted gain to each of the feature channels potentially constraints the level of object-specific feature binding. The following sub-chapters first describe the basic architecture of the model and then discuss characteristics and predictions of the model based on recent literature and the experimental findings of the current study.

## 3-step model of attention

**Figure 6:**
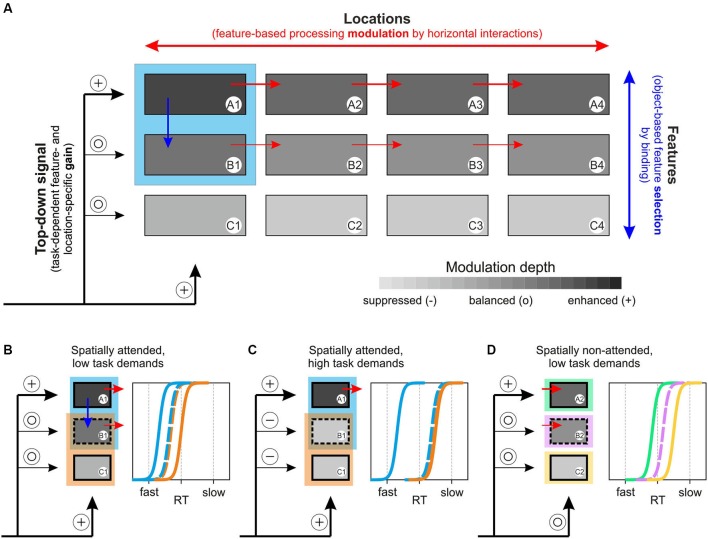
**3-step model of attention. (A)** Basic concept of the model, based on a task-dependent feature- and location-specific gain, object-specific feature selection by binding, and global, feature-specific processing modulation. The model builds on different feature channels (A–C), with each channel consisting of numerous modules to account for different locations (1–4). The blue rectangle represents an object at location 1, consisting of features A and B. A task-dependent top-down gain to each of the channels (symbolized by plus and open circle symbols to indicate modulation strength) sets the overall balance between the selected feature and other task-irrelevant features. Depending on this balance and on stimulus characteristics, vertical interactions between feature channels (blue arrow) represent object-specific binding dynamics (that may be attenuated if task-dependent top-down gain is set to induce suppression of task-irrelevant channels, cf. Figure 6C). Subsequently, features that are selected due to either task instructions (feature A) or binding dynamics (feature B) receive a global processing enhancement, mediated by horizontal interactions within feature channels (red arrows). **(B)** Predictions of RT distributions for two spatially overlapping objects at the spatial focus of attention, consisting of one feature that is unique to each of the objects and another that is shared by both objects, under low and **(C)** high task demands. **(D)** Predictions of RT distributions for three distant objects, consisting of either feature A, B, or C under stimulation conditions as those shown in Figure 6B. See main text for further explanation.

Several computational models of attention have been suggested previously, including Guided Search, Neural Theory of Visual Attention (NTVA), Selective Attention Model (SLAM), and others (for review: Itti and Koch, 2001; Wolfe and Horowitz, 2004; Bundesen and Habekost, 2005; Rothenstein and Tsotsos, 2008). The 3-step model of attention presented in the following is conceptual rather than computational, and represents a unified framework for feature- and object-specific effects of attention and their dependency on task requirements. The model consists of distinct feature channels (A to C in Figure 6A), each being represented by multiple modules to account for different locations in space (1 to 4 in Figure 6A), and a channel- and location-specific top-down input to these modules, specified by task requirements. The model assumes two forms of interaction, horizontally and vertically. Horizontal interactions are taking place between modules of the same feature channel and support enhanced processing of a selected feature at unattended locations. Vertical interactions are taking place between modules of different channels and essentially represent binding dynamics through which different features of an object are integrated. We propose that the actual strength of these vertical interactions determines the degree to which task-irrelevant features are subject to co-selection, depending on both the task-dependent, weighted top-down input to each of the feature channels and stimulus-specific characteristics. Attenuation or even suppression of these binding dynamics takes place if the top-down gain provides sufficient suppressive drive to those feature channels that process task-irrelevant information. Following feature selection (selected either directly as a consequence of task requirements or indirectly by object-specific binding processes), the strength of horizontal interactions then determines the degree to which these selected features are processed globally. In a nutshell, the model assumes task- and object-specific feature selection and object-independent global processing modulation of the selected features.

Consider an object at location 1, consisting of features A and B (blue square in Figure 6A), and a relatively undemanding task requiring attention to feature A. Step 1 of the model sets the top-down signal, which consists of a spatial, feature-unspecific selection of the task-relevant object location and a feature-specific selection of the task-relevant object feature A1. With low task demands, non-relevant feature channels will not be particularly suppressed, symbolized by open circles as inputs to channels B and C. Step 2 of the model consists of vertical interactions between different feature modules at the attended location, depending on two factors: 1) the top-down input to each of the feature channels as set in step 1; and 2) stimulus-dependent binding or grouping characteristics. In the example, binding is assumed to support object-specific feature integration, resulting in co-selection and enhanced processing of task-irrelevant object-feature B1 (blue arrow), but omitting feature C1. In the third step, the selected features receive a globally enhanced processing benefit, implemented by the horizontal interactions between modules of the same feature channels (red arrows). Hence, due to task requirements, feature A receives a global, strong processing enhancement and feature B receives a somewhat weaker (due to the absent top-down boost, cf. also Lu and Itti, 2005) but globally effective processing enhancement, too. Taken together, the model proposes a task- and object-specific selection of features (supported by binding dynamics and potentially constrained by top-down mediated suppression of task-irrelevant feature channels), and a global processing modulation of the selected features, i.e., not restricted to the initially attended object or location.

The model fully accounts for the experimental findings obtained in the current study and it makes numerous experimentally testable predictions, two of which are illustrated in Figures 6C,D and will be discussed below. First, for the results reported in this paper, Figure 6B illustrates our experimental situation by considering two objects, each consisting of one unique feature and another feature that is shared among both objects (illustrated by the partially overlapping blue and orange rectangles). The prediction from the model is that fastest RTs are to be expected for the unique, task-relevant feature A of the target object (blue), which receives a direct attention-dependent top-down boost, and slowest RTs for the unattended, unique feature C of the distractor object (orange). Yet, under low task demands (simple change detection under conditions of overt attention) the model predicts that the shared feature B will be subject to co-selection due to object-specific binding dynamics during step 2, but will be processed in a global, object-independent manner due to step 3, resulting in RTs that are to be the same regardless of whether this feature is tested at the blue or the orange object. Likewise, if attention is directed to the shared feature, RTs should be fastest for this feature, again independent of the object on which it is tested, and slowest for the other two features. The results of Experiments 1 and 2 for object-defining and shared features exactly confirm these predictions.

The basic characteristics of the model also predict results of previous studies that have been attributed to support either object- or feature-based attention. For example, O’Craven et al. (1999) reported that attending the motion of a face stimulus elicited higher activity not only in human motion-sensitive region MT+ but also in the fusiform face area (FFA), whereas activity in the parahippocampal place area in response to a spatially overlapping house stimulus was not affected. Considering low or moderate task demands and strong binding dynamics between motion and the high-level feature “face”, the 3-step model of attention predicts co-selection of the task-irrelevant feature “face” and enhanced processing in FFA, but no such effect for the feature “house”. Importantly, the model also predicts enhanced FFA activity in response to distant, task-irrelevant face stimuli, and to motion bound to the house stimulus. However, these conditions have not been tested in the study of O’Craven et al. (1999).

The results of O’Craven et al. (1999) were taken as evidence for object-based attention. Using essentially the same type of OFDA-paradigm, Treue and Martínez-Trujillo found evidence for feature-based attention by demonstrating that attending a specific feature of an object at a target location causes enhanced processing of that feature also at distant objects (Treue and Martínez Trujillo, 1999; Martínez Trujillo and Treue, 2004). This result is explained by the horizontal interactions of the model within feature channels. Importantly, as noted before, the model also predicts that under appropriate task and stimulus conditions another feature of the attended object may become subject to co-selection and enhanced processing. This condition was tested in a follow-up study by requiring attention to either the color or the motion of a moving object (Katzner et al., 2009). The authors found that attention-dependent effects of MT neurons were independent from the task at hand, supporting the assumption of co-selection of the task-irrelevant feature under experimental conditions for which results were otherwise consistent with feature-based attention.

Another line of evidence suggests that attention can also be directed away from known non-target features (Woodman and Luck, 2007; Arita et al., 2012). In the model, this can be achieved by setting a low weight or even a negative gain for task-irrelevant features, resulting in an advantage of other, not explicitly suppressed features. This effect would be in accordance with the finding that negative cues are effective, although not as powerful as positive ones (Arita et al., 2012).

## Influence of task demands and stimulus characteristics

A key-assumption of the model is that the strength of vertical interactions varies as a function of task difficulty, resulting from the weighted top-down input to the various feature channels. Thus, with higher task demands feature channels processing task-irrelevant information may become subject to active suppression (Figure 6C, symbolized by minus symbols as input for channels B and C), resulting in attenuation of binding these features with the selected feature, and thereby reducing or preventing their co-selection. For the example of RTs as a measure of attention-dependent processing modulation, higher task demands (as e.g., detecting a hardly visible feature change) will result in stronger suppression of task-irrelevant information and thus, in a right-ward shift of the RT distributions for the task-irrelevant target-object feature B. In the most extreme case, RTs may be as slow as those for the unattended feature C of the unattended object. In any case, RTs in response to the unattended feature B are predicted to still be independent from the object on which they are tested.

Experimental data from neurophysiological and neuroimaging studies support the assumption of a close relation between task demands and the specific form of attentional modulation. In monkey area V4, Spitzer et al. (1988) reported that neurons were more strongly modulated if monkeys had to detect an orientation difference of only 22.5° between sample and test stimuli as compared to a difference of 90°, and also found a corresponding behavioral improvement in discriminative abilities. Likewise, color-selective neurons in inferotemporal cortex were shown to be strongly modulated depending upon whether the task implied simple color categorization or a more demanding color discrimination (Koida and Komatsu, 2007). Notably, such task-related modulation of neuronal activity may be found as early as V1 (Chen et al., 2008), and has been reported for many areas throughout visual cortex in humans, including MT+ (Huk and Heeger, 2000).

Task demands may even cause a complete perceptual suppression of otherwise highly salient stimuli, as demonstrated by studies on inattentional blindness. A well-known example is the finding of overlooking the “gorilla-in-the-midst” (Simons and Chabris, 1999), but other studies showed that this complete recognition failure may also occur for less complex scenes and artificial stimuli, even if these were presented for prolonged times and moved through the center of gaze (Most et al., 2001). Active attention-dependent inhibition was demonstrated by Slotnick et al. (2003), reporting significant suppression of activity at locations distant to the attended object, in both striate and extrastriate visual areas. Other studies investigated the processing fate of different object features and found evidence for both, co-selection and suppression, suggesting that feature-directed attention may act through a combination of facilitatory and inhibitory mechanisms (Fanini et al., 2006; Xu, 2010). Importantly, whether an irrelevant object feature was selected or blocked depended upon task requirements or attentional load. Active inhibition was evident only if the task induced a strong response conflict, whereas it was absent otherwise (Fanini et al., 2006), or as a function of the target-feature encoding load (Xu, 2010). In addition, effective filtering of a task-irrelevant feature has been shown to increase with learning (Gál et al., 2009), underlining the dynamic nature of feature selection and feature suppression.

Task demands may also vary with stimulus characteristics. Mayer and Vuong (2012) recently showed that changes to unattended motion or color of a stimulus did not affect a subject’s performance, but changes to unattended shape did. These results provide direct evidence for stimulus-inherent properties influencing the degree to which irrelevant object features of the attended object can be effectively suppressed. In turn, they also suggest that stimulus properties influence the degree to which irrelevant information is bound to the relevant information. Such spreading of attention was shown by previous behavioral (Egly et al., 1994; Richard et al., 2008) and single-cell studies (Roelfsema et al., 1998, 2004), demonstrating that unattended locations receive a processing enhancement when these were located on the same coherent object than the attended location, as compared to equally distant but unbound locations. Gestalt cues like collinearity, color similarity, and common fate similarly influence attentional spreading towards irrelevant locations (Wannig et al., 2011). These authors demonstrated increased V1 firing rates in response to a spatially unattended stimulus depending on its Gestalt similarity to a stimulus at the attended location. The results were taken as support for the concept of incremental grouping, which builds on labelling of feature-selective neurons, e.g., by enhanced activity (Roelfsema et al., 2000; Roelfsema, 2006). Accordingly, if applied to the framework presented here, task- and binding-mediated enhancement or suppression of feature-selective neurons would determine the degree to which these are labelled and thus directly influences potential co-selection of task-irrelevant object features.

## Parallel, feature-specific processing enhancement

Due to step 3 of the model, another key-assumption is that all selected features gain a global, i.e., spatially independent processing enhancement, no matter whether they were selected by task instructions or as a result of object-specific binding dynamics. Thus, when tested on a distant object, the model not only predicts a processing benefit for the attentionally selected object-feature, but for co-selected features as well. In Figure 6D, each of the features A to C is tested on a different object at the unattended location 2. The model predicts that basic relations between RT distributions as observed at the attended object (cf. Figure 6B) should be preserved in the periphery, even though shifted to the right due to absent spatial attention (indicated by an open circle for location 2). Thus, the attended feature A receives fastest RTs and the co-selected feature B receives somewhat slower RTs, but still faster than those of the unselected feature C.

By predicting global processing enhancement of all selected features, the model necessarily implies that attention can be divided to multiple features at the same time, as also suggested by the results of the current study showing reduced RTs not only for the cued feature but also in response to the uncued, co-selected feature. This finding is in accordance with previous research indicating that parallel processing of two attended features may occur without costs in accuracy as compared to processing only one (Bonnel and Prinzmetal, 1998; Tsujimoto and Tayama, 2004). Interestingly, dual-task performance involving feature values defined in the same dimension (form, color, motion) was reported to be indistinguishable from dual-task performance involving features from different dimensions (Lee et al., 1999). The most direct proof of divided feature-directed attention has been provided by recent EEG studies using frequency-tagged, steady-state visual evoked potentials (Andersen et al., 2008, 2013). It was not not only shown that attention can indeed be directed to two different features at the same time, but furthermore that facilitation of these features can be observed throughout the visual field even if task demands would favor a spatially restricted processing enhancement (Andersen et al., 2013).

Consistent with this and our own findings, another EEG study recently showed that also the neuronal representation of task-irrelevant features may be globally enhanced (Boehler et al., 2011). The authors investigated the ERP response to a distractor object, located in the hemi-field opposite to the target. Even though the object was irrelevant to the task and located outside the spatial focus of attention, its neuronal representation was modulated depending on the similarity of distinct features between distractor and target. Specifically, if the distractor contained a color that was also present in the target, the ERP response showed a characteristic modulation as compared to the situation when both objects were made of different colors. Interestingly, this irrelevant-feature effect arose about 80 ms later in time than the attentional effect at the target object, a modulation of the N2pc component (being associated with the allocation of attention and also linked to feature selection (Luck and Hillyard, 1994; Eimer, 1996; Hopf et al., 2004)). The authors interpreted this result as to indicate spreading of attention towards other objects outside the spatial focus of attention, as previously been also suggested by studies showing object-based response compatibility effects at distractor items (Chen and Cave, 2006), and an influence of categorical similarity (Kravitz and Behrmann, 2011). In the context of our model, this feature-depending modulation of the distractor is in accordance with representing step 3 of the model—a global, feature-based enhancement of those features that were selected during step 1 and 2. Further experimental results in accordance with this notion come from a recent fMRI study demonstrating global enhancement of co-selected, task-irrelevant features bound to the target feature of the attended object (Lustig and Beck, 2012). Notably, this spreading of attention from the target to the distractor object not only occurs under conditions of covert attention, as shown by Boehler et al. (2011) and Lustig and Beck (2012), but even when objects are presented at the spatial focus of attention, as indicated by the results of the current study.

## Top-down adjustment

The primary purpose of the model is to suggest a simple, unique framework to account for (1) the experimental results obtained in our experiments; and (2) experimental findings from previous studies that have been attributed to either feature-based or object-based attention. In its current version, the model does not distinguish between features of the same (red, green) or different dimensions (color, motion). There is good evidence that attending a specific feature dimension may affect processing of all features in that dimension (Found and Müller, 1996; Weidner et al., 2002; Gramann et al., 2007; Schubö and Müller, 2009; Gramann et al., 2010), thus posing the constraint that processing of task-irrelevant features may be different depending on whether these are defined in the same or a different dimension. However, such a distinction can easily be incorporated into the model by splitting the top-down gain into two factors, one concerning the feature dimension and the other concerning the specific feature attribute. If all other characteristics of task and stimuli are kept constant, the model allows for predicting the relative size of attentional effects as a function of the dimension to which the task-irrelevant object features belong.

A possible candidate structure as the source of this task-dependent top-down signal is the prefrontal cortex (PFC), a region involved in the executive control of behavior and the current task set (Sakai, 2008). Many neurons in PFC exhibit a strong rule-dependency regarding spatial and featural decisions, and the acquisition and implementation of the current task context has been suggested to constitute a main function of PFC (Sakagami and Niki, 1994; White and Wise, 1999; Assad et al., 2000). Interestingly, in the context of the current study, the activity of a significant fraction of neurons in PFC has been demonstrated to exhibit task-dependent selectivity for both the behaviorally relevant features motion and color (Lauwereyns et al., 2001).

## Concluding remarks and summary

The current study utilized 2-dimensional cues indicating the prospective target object and target feature, and RT as a measure for behavioral performance. The significant dependencies between the information provided by the cue and the respective RT distributions were interpreted as representing feature- and object-based attentional selection, and were integrated into a 3-step model of attention acting on the early processing of visual stimuli. Yet, opposed to this assumption, differences in RT do not necessarily indicate an influence on visual processing, but potentially might also be due to other factors, as e.g., a task-specific response set (cf. for discussion: Taya et al., 2009). However, the strong evidence provided by several neurophysiological studies revealing the influence of both object- and feature-based attention on neuronal responses in early visual cortex (Roelfsema et al., 1998; Treue and Martínez Trujillo, 1999; McAdams and Maunsell, 2000; Wannig et al., 2007; David et al., 2008; Katzner et al., 2009; Zhou and Desimone, 2009; Wannig et al., 2011; Chen et al., 2012), and the correlation between attention-dependent modulation of neuronal activity in early visual cortex and behavioral RT of the animal (Cook and Maunsell, 2002; Womelsdorf et al., 2006b; Herrington and Assad, 2009; Galashan et al., 2013) provides strong support for relating the RT effects observed in our psychophysical experiments to modulations during early visual processing. The RT measurements and their strong dependence on the feature- and object-specific cueing condition suggest the co-existence of attention-dependent effects commonly attributed to different frameworks of attention. Our model provides a new conceptual framework into which existing theories of neuronal implementations of attention may be incorporated, as e.g., the feature-similarity gain model (Treue and Martínez Trujillo, 1999; Martínez Trujillo and Treue, 2004), or the incremental grouping hypothesis (Roelfsema et al., 2000; Roelfsema, 2006). By assuming a top-down gain adjustment, task- and object-specific binding dynamics, and a global feature-specific response modulation, the model not only explains our own experimental results within a single, coherent framework, but also allows for the unification of a vast amount of experimental data that were usually taken as support for either object- or feature-based attention. Future research for testing predictions of the model regarding the influence of task demands and object-specific binding dynamics on the proposed global nature of processing modulation will reveal benefits and limits of the model, and new insights in the complex interdependencies of various attention- and task dependent mechanisms.

## Author contributions

Detlef Wegener, Maike Kathrin Aurich, Fingal Orlando Galashan, and Andreas Kurt Kreiter designed research, Fingal Orlando Galashan contributed stimulation programs, Detlef Wegener and Maike Kathrin Aurich acquired data, Detlef Wegener analyzed data, and Detlef Wegener wrote the paper.

## Conflict of interest statement

The authors declare that the research was conducted in the absence of any commercial or financial relationships that could be construed as a potential conflict of interest.
